# A Bidirectional Long Short-Term Memory Model Algorithm for Predicting COVID-19 in Gulf Countries

**DOI:** 10.3390/life11111118

**Published:** 2021-10-21

**Authors:** Theyazn H. H. Aldhyani, Hasan Alkahtani

**Affiliations:** 1 Applied College in Abqaiq, King Faisal University, P.O. Box 400, Al-Ahsa 31982, Saudi Arabia; 2 College of Computer Science and Information Technology, King Faisal University, P.O. Box 400, Al-Ahsa 31982, Saudi Arabia; hsalkahtani@kfu.edu.sa

**Keywords:** Bi-LSTM, deep learning, time series model, COVID-19, Gulf countries

## Abstract

Accurate prediction models have become the first goal for aiding pandemic-related decisions. Modeling and predicting the number of new active cases and deaths are important steps for anticipating and controlling COVID-19 outbreaks. The aim of this research was to develop an accurate prediction system for the COVID-19 pandemic that can predict the numbers of active cases and deaths in the Gulf countries of Saudi Arabia, Oman, the United Arab Emirates (UAE), Kuwait, Bahrain, and Qatar. The novelty of the proposed approach is that it uses an advanced prediction model—the bidirectional long short-term memory (Bi-LSTM) network deep learning model. The datasets were collected from an available repository containing updated registered cases of COVID-19 and showing the global numbers of active COVID-19 cases and deaths. Statistical analyses (e.g., mean square error, root mean square error, mean absolute error, and Spearman’s correlation coefficient) were employed to evaluate the results of the adopted Bi-LSTM model. The Bi-LSTM results based on the correlation metric gave predicted confirmed COVID-19 cases of 99.67%, 99.34%, 99.94%, 99.64%, 98.95%, and 99.91% for Saudi Arabia, Oman, the UAE, Kuwait, Bahrain, and Qatar, respectively, while testing the Bi-LSTM model for predicting COVID-19 mortality gave accuracies of 99.87%, 97.09%, 99.53%, 98.71%, 95.62%, and 99%, respectively. The Bi-LSTM model showed significant results using the correlation metric. Overall, the Bi-LSTM model demonstrated significant success in predicting COVID-19. The Bi-LSTM-based deep learning network achieves optimal prediction results and is effective and robust for predicting the numbers of active cases and deaths from COVID-19 in the studied Gulf countries.

## 1. Introduction

Corona disease of 2019 (COVID-19) is a new respiratory disease that first reported in Wuhan City, Hobi Province, China. This disease differs from other coronaviruses (CoV) that are spread between humans in that it causes the symptoms of reproductive extinction of CoV [1]. On 21 January 2020, the World Health Organization (WHO) suggested that there was a possible sustained human-to-human transmission of the novel COVID diseases [2]. On 31 January 2020, the WHO declared a global emergency, and on 11 March, the disease was recognized as a global pandemic [3].

Current estimates suggest that the time of the incubation of the disease is 14 days after exposure. Symptoms include a rise in temperature and trouble breathing, especially if these symptoms appear 14 days from the date of travel to China or other countries with confirmed COVID-19 cases. Suspected patients are given instructions and treatments by healthcare providers before attending hospital or presenting for laboratory examinations.

Currently, the WHO has approved some specific vaccines for COVID-19 [4,5]. In the past, many pandemics, such as those stemming from severe acute respiratory syndrome (SARS), were controlled and halted by using conventional control measures, including patient isolation and travel restrictions. Presently, these measures are being implemented in most countries to reduce the spread of the COVID-19 outbreak [6,7]. In fact, having methods that enable reliable prediction of the spread of COVID-19 would be of great benefit in persuading the public that it is crucial to adhere to these measures [8,9].

On 28 January 2020, the United Arab Emirates (UAE) Ministry of Health and Community Protection announced a diagnosis of the new COVID-19 for one family member coming from Wuhan City in the People’s Republic of China. This was the first confirmed case in the Gulf countries, and it was followed by a rapid increase in the confirmed cases in the region. On 20 April 2020, Saudi Arabia topped the list of Gulf states in terms of the number of people infected with the Middle East respiratory syndrome (MERS)-CoV, with 10,484 cases and 103 deaths, followed by the UAE with 7265 cases and 43 deaths, Qatar with 6015 cases and 9 deaths, Bahrain with 1895 cases and 7 deaths, Kuwait with 1995 cases and 6 deaths, and Oman with 1410 and 7 deaths, according to the latest statistics prepared by Gulf Online. The world is currently facing rapid developments related to the outbreak of COVID-19, and Gulf countries are seeking precautionary and preventive measures to reduce the spread of the diseases and mitigate its effects. Such important measures have had an impact on the business environment and the overall output of society. The Gulf countries have put in place packages of resolutions and measures to mitigate the economic effects of the pandemic to help the business sector overcome it with the least amount of damage. Table 1 shows that such circumstances raise numerous inquiries about the potential effects of this epidemic on financial reports, and as highlighted by the Saudi Association of Chartered Accountants, the international standards of the financial report adopted in Saudi Arabia are based on principles that require the administration to work hard. For the sake of obtaining information about the influence of COVID-19 on the population of particular countries and predicting the number of possible cases and expected dates for the coronavirus pandemic to end in these countries, we proposed a machine learning model that can be controlled on Cloud Data Centers (CDCs) to provide precise information on predicting the spread of the disease. This will help in monitoring the response of the governments and citizens.

The availability of accurate models for predicting outbreaks provides valuable information on diseases that are likely to spread and their consequences. This collected information gives governments and other organizations the chance to propose new policies that can help meet the threats posed by these pandemics [10]. The COVID-19 pandemic has resulted in the infection of more than 162 million people and the registration of more than 3.3 million deaths around the world. Compared with other outbreaks, COVID-19 has shown a nonlinear nature and different features that raise questions about the effectiveness of the available standard models [11]. Many variables, including differences in behaviors among individuals in diverse geographical areas and differences in the policies for controlling the pandemic, play significant roles in decreasing the accuracy of the existing models [12]. Thus, current models face some challenges that interfere with the precision of the obtained results. New models have been developed to cope with these challenges by adopting additional assumptions, such as the effects of quarantines, social distancing, and lockdowns [13,14,15].

## 2. Related Work

Several prediction systems have been proposed by different researchers for predicting the spread of COVID-19, e.g., [16,17,18,19]. Machine learning algorithms for predicting COVID-19 have been described [20,21,22]. Other researchers [23,24] have used evolution-based convolutional neural networks, while still others [25,26] have adopted transmission models for the prediction of COVID-19. An agent-based method has also been invoked [27] to predict influenza epidemics in China via a search query [28]. Researchers have also used Bayesian regression models [29,30] or have attempted to investigate the evolution of COVID-19 to find the causes of the disease [31,32]. Recently, social media have been exploited to find the correlation between clinical data and queries submitted by people with regard to the transmission of the infection [33,34,35].

Earlier studies have focused on predicting the transmission of other epidemics, such as SARS [36], influenza [37], and dengue fever [38]. Since the beginning of the COVID-19 pandemic, many researchers have attempted to predict the number of confirmed cases and deaths in particular countries in an effort to decrease the spread of the disease. The systems proposed in these studies have proven useful for governments, official health organizations, and the WHO. For example, health surveillance systems can be developed using Twitter data to predict infectious diseases [39].

Some studies have proposed deep learning algorithms to predict infectious diseases [40,41], as these algorithms can be used to analyze and predict big data from healthcare datasets [42,43]. Xu et al. [40] used a deep learning approach and a statistical model, such as the autoregressive integrated moving average (ARIMA) model, for forecasting purposes and noted that the deep learning algorithm achieved better performance [40]. Aldhyani et al. [44] introduced an adaptive fuzzy inference neural network (ANFIS) model to forecast chronic diseases using Google search queries. Theyazn et al. [45] used a soft computing algorithm to classify chronic diseases. Kang et al. [46], Yuan et al. [47], and Philip et al. [48] developed surveillance systems that could forecast influenza outbreaks based on internet data. Milinovich et al. [49] developed a surveillance system to predict infectious diseases by employing search data from an Australian engine search, while. Cook et al. [50] used machine learning to predict COVID-19 methodology.

COVID-19 modeling and prediction are highly significant undertakings for determining the imaginable future impact of the disease. Developing a model for the spread and effects of COVID-19 can be tremendously important for understanding the COVID-19 impact [51]. In fact, artificial intelligence and traditional statistical models can help in modeling and forecasting COVID-19 [52,53,54,55,56,57]. In the current research work, the main goal was to achieve an accurate prediction model using a deep learning algorithm.

Zeroual et al. [58] predicted infectious cases in four countries by applying five models: recurrent neural network, LSTM, Bi-LSTM, gated recurrent unit, and variational autoencoder algorithms. Gupta et al. [59] implemented susceptible–exposed–infectious–removed and regression models to forecast COVID-19 cases in India. Shahid et al. [60] proposed ARIMA, SVR, LSTM, and Bi-LSTM to predict COVID-19 instances. Yadav et al. [61] introduced support vector machine (SVM), naïve Bayes (NB), LR, decision tree (DT), random forest (RF), and LSTM to predict confirmed COVID-19 cases based on data from different countries. Rustam et al. [62] also used different models, such as SVM and exponential smoothing (ES), to forecast harmful factors that promote COVID-19 spread. Other authors have used different deep learning neural network algorithms, such as the long short-term memory model (LSTM) [61,62,63] and a gated recurrent unit (GRU)-based model [64], for predicting various time series applications. The robustness of each model relies on the estimation errors and the time costs.

Jelena et al. [65] used the regression method for modeling and predicting COVID-1 infection. They also discussed the use of machine learning and evolutionary computing (EC) methods with regression. Their dataset was gathered from various regions to examine their proposed system, which was used to predict active, confirmed, and active cases. Evaluation of the results of the proposed system using the mean square error (MSE) and root mean square error (RMSE) metrics revealed the system score with the highest percentages of RNN (95.1), ARIM (0.95), and MPL (98%) with respect to R2. Isra et al. [66] proposed Holt–Winters and ARIMA models to predict COVID-19 in Saudi Arabia from 24 March 2020 to 5 April 2021. They noted that the ARIMA model gave a smaller RMSE (1225.9), mean absolute error (MAE) (921.3), and mean percentage error MAPE (25.0). Khaled et al. [67] proposed the use of an ARIMA model and a susceptible–infected–recovered (SIR) model to predict the spread of COVID-19 in Saudi Arabia in the time interval from 2 March 2020 to 30 June 2020. Their application of an ARIMA model with different lags (1,0,2), (1,1,1), (1,1,3) showed that an ARIMA model with lag (1,1,1) was better than the others and gave scores of R2 (0.96) and RMSE (341) in that period. Nahla et al. [68] presented two deep learning approaches (LSTM and GRU) to predict COVID-19 infection in Egypt, Kuwait, and Saudi Arabia from 1 May 2020 to 12 June 2020. The empirical results showed that the LSTM model achieved the highest percentages for the evaluation metrics MAPE (0.445), RMSE (29.8), and MAE (28.0). Sina et al. [69] presented a study comparing SIR and susceptible–exposed–infectious–removed (SEIR) models to forecast COVID-19 based on a dataset collected from Italy, China, the USA, Iran, and Germany with a time period of 30 days. They used RMSE to test the obtained results of the proposed system and obtained RMSE values of 1020 for Italy, 2524 for China, 1267 for Iran, 22.35 for the USA, and 55.14 for Germany. Gergo et al. [70] used the adaptive network-based fuzzy inference system (ANFIS) model to predict COVID-19 infections with datasets collected from the Hungarian surveillance system over a period of nine days. Use of the MAPE, RMSE, and determination coefficient metrics to test and evaluate the proposed system revealed that the proposed system can serve as an alternative model for a strategy to control the spread of COVID-19.

The Bi-LSTM model, which forecasts the maximal number of confirmed cases that will contract the disease per country in a specific time interval and the maximal number of death cases per country, has been proposed to predict COVID-19 cases in the Gulf countries. The main objectives of this research were to present an advanced Bi-LSTM artificial intelligence model to forecast COVID-19 in the Gulf countries, use a validation method to confirm the reliability of the Bi-LSTM model for estimating COVID-19, and include COVID-19 datasets from more than one country to test the robustness of the developed Bi-LSTM model. The novelty of this study is that it describes the development of a system that can assist government agencies and medical personnel to be prepared for forthcoming pandemics and to ensure greater readiness in the healthcare systems.

## 3. Materials and Methods

This section presents the Bi-LSTM network for predicting COVID-19. The present research used real COVID-19 data collected from the website Our World in Data. The Bi-LSTM algorithm was proposed to predict the number of active cases and deaths in the Gulf countries of Saudi Arabia, Oman, the United Arab Emirates (UAE), Kuwait, Bahrain, and Qatar.

### 3.1. Dataset Description

**Figure 1:**
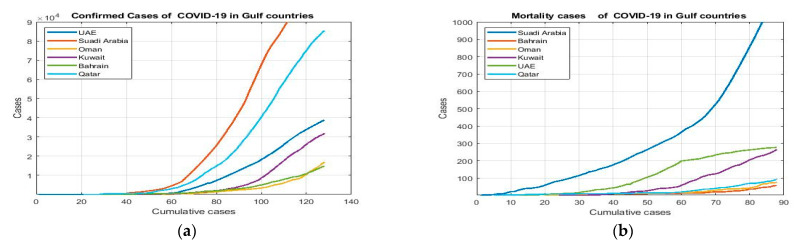
COVID-19 datasets for Gulf countries: (**a**) confirmed cases; (**b**) deaths.

The research data were gathered from the publicly available repository https://ourworldindata.org/coronavirus-testing (accessed on 15 July 2020). The available repository contains updated data for COVID-19 cases around the world. In this research, we focused on the Gulf countries of Saudi Arabia, Oman, the UAE, Kuwait, Bahrain, and Qatar. We collected confirmed COVID-19 cases by different start dates, as mentioned in Table 2, to the end date of 8 June 2020. Table 3 summarizes the simple dataset of death cases with different time intervals to 8 June 2020. Each country has a different time interval, as presented in Figure 1. The *x*-axis values represent the numbers of instances for each country, while the *y*-axis values represent the range of values of daily cases.

### 3.2. Normalization

A min–max method was used to transform the data to values between zero and one, as scaling the data can improve the system for predicting COVID-19. The two main advantages of scaling are to avoid instances of greater numeric ranges dominating those with smaller numeric ranges and to prevent numerical difficulties during the prediction. The transformation is accomplished as follows (Equation (1)):

(1) $$z_{n}=\frac{x-x_{min}}{x_{max}-x_{min}}\left(New_{max_{x}}-New_{min_{x}}\right)+New_{min_{x}}$$

where $x_{min}$ is the minimum of the data, and $x_{max}$ is the maximum of the data. $New_{min_{x}}$ is the minimum number zero, and $New_{max_{x}}$ is the maximum number one.

### 3.3. Bidirectional Long Short-Term Memory Algorithm (Bi-LSTM)

Memory units in long short-term memory (LSTM) can ultimately carry the results from observation data X into the prediction of Y. The process of implementation occurs in only one direction, forward, which neglects the backward connection and makes the system less efficient. This drawback is overcome by performing the data-training phase in a Bi-LSTM system in two sequential directions: forward and backward. This improves the performance of the model [71,72,73,74,75,76,77]. Bi-LSTM was used for forecasting along coastal areas of Queensland, Australia [78], and a developed Bi-LSTM model was used for COVID-19 cases in Japan [79]. Yuchao et al. [80] proposed a Bi-LSTM model to predict ship roll. Program code is variable in this link: https://github.com/Theyazn/A-Bidirectional-Long-Short-Term-Memory-Model-Algorithm-for-Predicting-COVID-19- (accessed on 17 September 2021).

**Figure 2:**
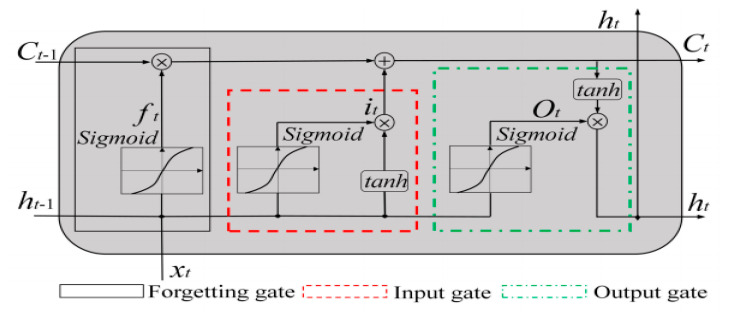
Structure of the LSTM model.

**Figure 3:**
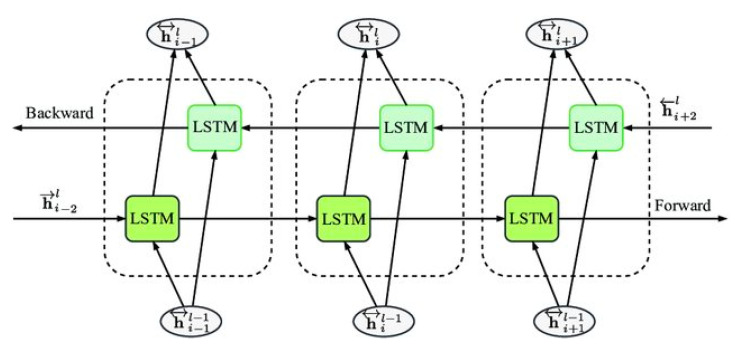
Structure of the bidirectional long short-term memory algorithm.

The framework of the long short-term memory model is presented in Figure 2. The structure of Bi-LSTM is displayed in Figure 3. Table 4 shows the significant parameters of the Bi-LSTM algorithm for predicting COVID-19 in the Gulf countries. 

These parameters were appropriate for the Bi-LSTM algorithm to predict COVID-19. (Equations (2)–(4))

(2) $$f_{t}=\;\sigma \;(W_{ef}X_{t}+W_{ef}h_{t-1}+W_{cf}C_{t-1}+b_{f})\;$$

(3) $$i_{t}=\;\sigma \;(W_{xi}X_{t}+W_{hi}h_{t-1}+W_{ci}C_{t-1}+b_{i})\;$$

(4) $$o_{t}=\;\sigma \;(W_{xo}X_{t}+W_{ho}h_{t-1}+W_{co}C_{t-1}+b_{o})\;$$

where

$i_{t}$,$\;f_{t}$ and $o_{t}$: are input gate, forget gate, and output gate, respectively;

$X_{t}$: is input data;

$W_{ef},\;W_{ho},\;W_{xo},W_{hi},$$\;W_{cf},$$\;W_{co}$ and $W_{ci}$: are weighted neural cell;

$b_{o}$: is bias;

$h_{t-1}$: short memory vector;

$C_{t-1}$: long memory vector.

Using the input gate and forget gate, the LSTM model uses the validated input data to update the formula into (Equations (5) and (6))

(5) $$C_{t}=\;\sigma \;(f_{t}c_{t-1}+i_{t}\tanh(W_{xc}X_{t}+W_{hc\;}h_{t-1}+b_{c}),\;$$

(6) $$h_{t}=O_{t}\times \tanh(C_{t}),\;$$

where

$C_{t}:$ cell gate;

$h_{t}$: is a hidden state to correspond to input data.

All gates depend on the size of the data. W is the weight matrices between the cells. The bias values for each layer are designated by b, and the sigmoid function *σ* and hyperbolic tangent function are used as activation functions. The activation function formulas are represented in Equations (7) and (8).

(7) $$Sigmoid=\frac{1}{1+e^{-z}}$$

(8) $$\tanh=\frac{e^{z}-e^{-z}}{e^{z}-e{-}^{z}}$$

where z is the input data.

### 3.4. Model Evaluation Criteria

The Bi-LSTM algorithm is evaluated and tested using the evaluation metrics. Five statistical analyses (the MSE, RMSE, mean absolute error [NRMSE], and Spearman’s correlation coefficient squared [R^2^]) are applied to evaluate the Bi-LSTM algorithm. These metrics are expressed as follows (Equations (9)–(12)):

(9) $$MSE=\frac{1}{N}\;\overset{n}{\underset{i=1}{\sum }}{\left(x_{i}-{\bar{x}}_{i}\right)}^{2},\;$$

(10) $$RMSE=\sqrt{\frac{1}{N}\sum_{i=1}^{n}{\left(x_{i}-{\bar{x}}_{i}\right)}^{2}},\;$$

(11) $$\mathrm{NRMSE}=\frac{\sqrt{\frac{1}{N}\sum_{i=1}^{n}{\left(x_{i}-{\bar{x}}_{i}\right)}^{2}}\;}{\bar{x}},\;$$

(12) $$R^{2}=1-\frac{\sum {\left(x_{i}-{\bar{x}}_{i}\right)}^{2}}{\sum {\left(x_{i}-{\bar{x}}_{i}\right)}^{2}\;}\;\times \;100\%,$$

where $x_{t}\;$represents real data, ${\bar{x}}_{t}\;$represents prediction values, and *N* is the total number of samples.

## 4. Results

The Bi-LSTM model was suggested to forecast COVID-19 in the Gulf countries. The performance of the Bi-LSTM algorithm was evaluated and examined using a real dataset collected from the public world repository. The proposed system was validated by dividing the datasets into 80% training data and 20% testing data.

Table 5 presents the training results of the Bi-LSTM model for predicting the numbers of confirmed COVID-19 cases in the Gulf countries. The empirical results were compared using the MSE, RMSE, NRMSE, and R^2^ evaluation metrics. The proposed system achieved good accuracy in predicting the number of confirmed cases in Qatar during the COVID-19 pandemic. The prediction results obtained from the training dataset for Oman were MSE = 4.4217×10^−5^, RMSE = 0.00664, and NRMSE = 0.00372.

Table 6 summarizes the training results of the Bi-LSTM model for predicting deaths from COVID-19 in the Gulf countries in different periods. The mortality rate in the Gulf countries is low because governments took isolation precautions and locked down cities for long periods. The Gulf countries have fewer deaths than other countries around the world. We proposed a robust deep learning algorithm, the Bi-LSTM model, for predicting mortality rates in these countries. The prediction results of the Bi-LSTM model for predicting deaths in Saudi Arabia were superior, with values of 3.8242 × 10^−5^, 0.00618, and 0.036 for the MSE, RMSE, and NRMSE, respectively. The prediction value and actual values were very close.

**Figure 4:**
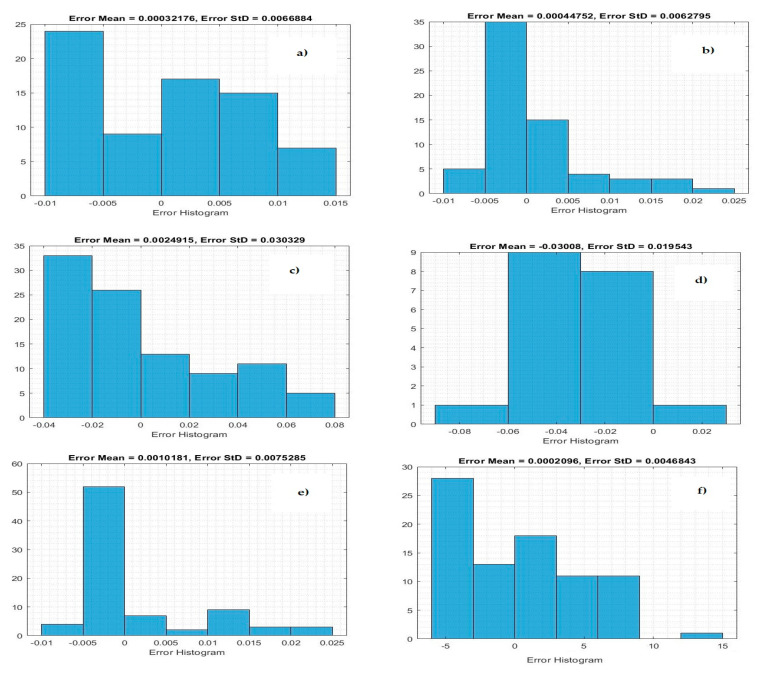
Histogram error of the training data for predicting numbers of confirmed cases: (**a**) Saudi Arabia, (**b**) Oman, (**c**) United Arab Emirates, (**d**) Kuwait, (**e**) Bahrain, and (**f**) Qatar.

**Figure 5:**
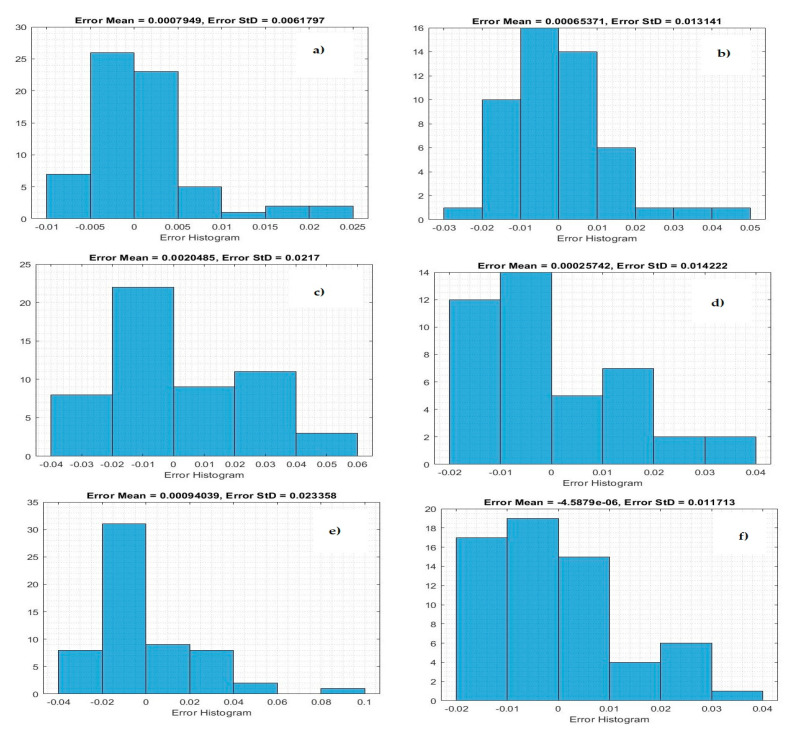
Histogram error of training data for predicting mortality: (**a**) Saudi Arabia, (**b**) Oman, (**c**) United Arab Emirates, (**d**) Kuwait, (**e**) Bahrain, and (**f**) Qatar.

Figure 4 demonstrates the histogram error of the predicted values of the confirmed case of COVID-19 at the training phase. The error histogram is employed to calculate the differential between the prediction values and observation values. We derived the maximum error from prediction values and target values. While the *x*-axis values represent scaling values, the *y*-axis shows the number of times that values occurred within the scaling values set (*x*-axis), representing the instances of values obtained by the proposed system. The minimum error was reported as 0.000020 for predicting confirmed cases in Qatar, and the overall histogram errors are much smaller. Figure 5 shows the histogram error of the predicted values for mortality cases of COVID-19 at the training phase for the Gulf countries. The minimum error was reported as 0.0006530 for predicting confirmed cases in Oman, but the overall histogram errors were much smaller.

### Validation of the Proposed Model

This section presents the validation of the Bi-LSTM model to forecast the confirmed cases and mortality rate in the Gulf countries of Saudi Arabia, Oman, the UAE, Kuwait, Bahrain, and Qatar. We validated the proposed Bi-LSTM model using 20% of the data as a testing set. The testing results of the Bi-LSTM model were proposed to validate the obtained results. Table 7 demonstrates the prediction results as a testing stage of the Bi-LSTM model for predicting confirmed cases in the Gulf countries. The Bi-LSTM model provided the highest prediction of the confirmed cases in the UAE and the lowest prediction of confirmed cases in Bahrain.

The prediction results at the testing phase of the Bi-LSTM model to forecast the number of confirmed cases in Saudi Arabia were 0.00014, 0.01222, and 0.0163 for MSE, RMSE, and NRMSE, respectively; for Oman, they were 0.00161, 0.0401, and 0.0651, respectively; for the UAE, they were 0.001518, 0.03886, and 0.0510, respectively; for Kuwait, they were 0.001518, 0.03886, and 0.05103, respectively; and for Bahrain, they were 0.00111, 0.01053, and 0.01453, respectively. The testing values and predicted values were very close, indicating that the prediction errors were low. The system showed significant ability to predict confirmed cases in Bahrain and Saudi Arabia data at the testing stage.

**Figure 6:**
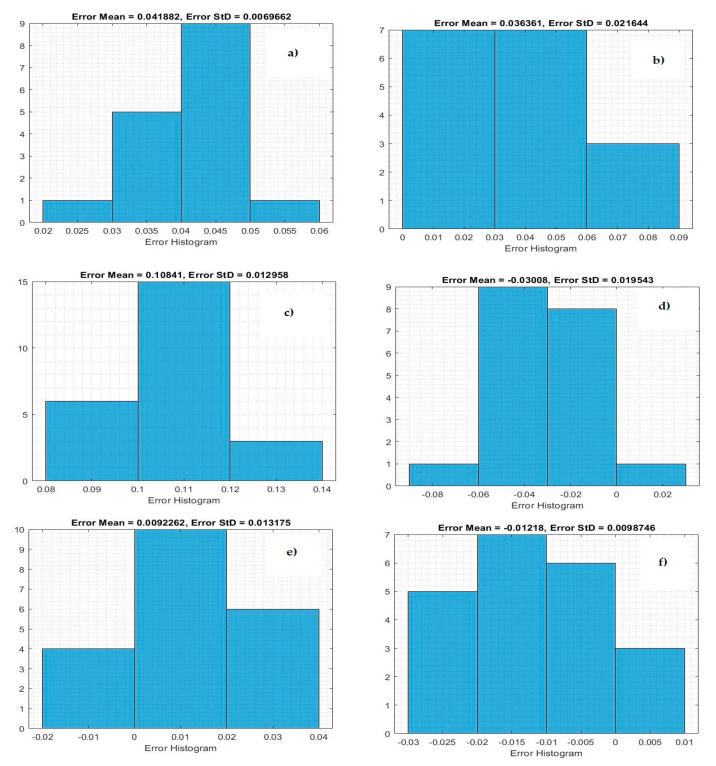
Histogram error of the testing phase for predicting confirmed cases: (**a**) Saudi Arabia, (**b**) Oman, (**c**) United Arab Emirates, (**d**) Kuwait, (**e**) Bahrain, and (**f**) Qatar.

Table 8 shows the prediction results of the proposed system to predict mortality cases as a testing stage in Gulf countries. The proposed system has achieved fewer prediction errors in predicting mortality cases in Kuwait, with MSE = 0.00152, RMSE = 0.01233, and NRMSE = 0.0145. Figure 6 shows histogram metrics for predicting the confirmed case at the testing phase. The system achieved minimum errors in the testing phase for predicting confirmed cases in Bahrain, with an average error of −0.01218.

**Figure 7:**
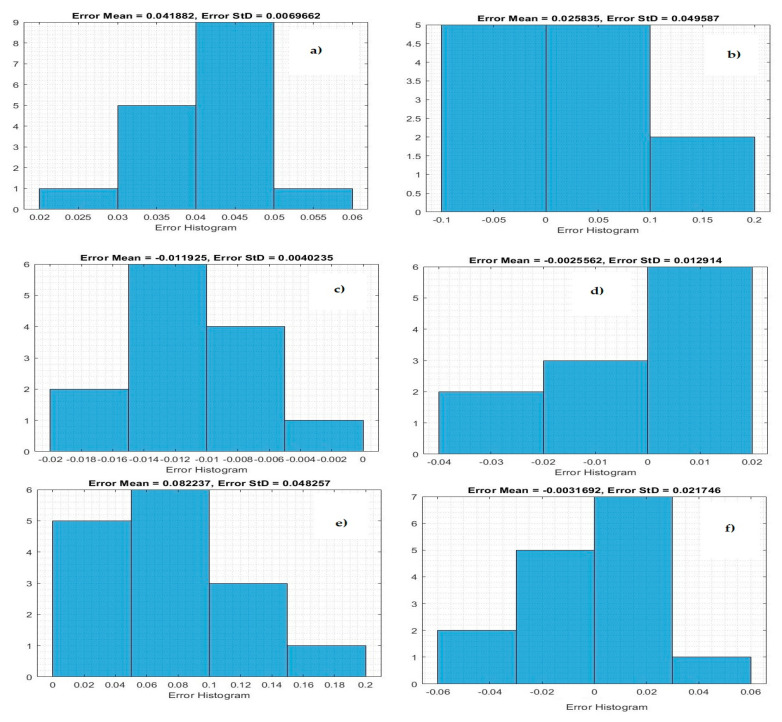
Histogram error of the testing phase for predicting mortality cases: (**a**) Saudi Arabia, (**b**) Oman, (**c**) United Arab Emirates, (**d**) Kuwait, (**e**) Bahrain, and (**f**) Qatar.

Based on the obtained results, the proposed model showed the best prediction results in the testing stage for forecasting the number of deaths in Kuwait. The testing results of the Bi-LSTM model for predicting the death toll in Kuwait were 0.0001512, 0.01233, and 0.014 for the MSE, RMSE, and NRMSE, respectively. Figure 7 shows the histogram prediction performance of the Bi-LSTM model in forecasting mortality in the Gulf countries. The average error of the proposed system is much smaller for Kuwait. The proposed model shows good performance.

A comparison between the target values and predicted values of confirmed COVID-19 in the Gulf countries is presented in Table 9. The percentages of the mean errors were very low after using min–max normalization methods. The proposed system was tested by taking 20% of the observation data for testing, and the results indicate the high robustness of the developed model for predicting COVID-19.

**Figure 8:**
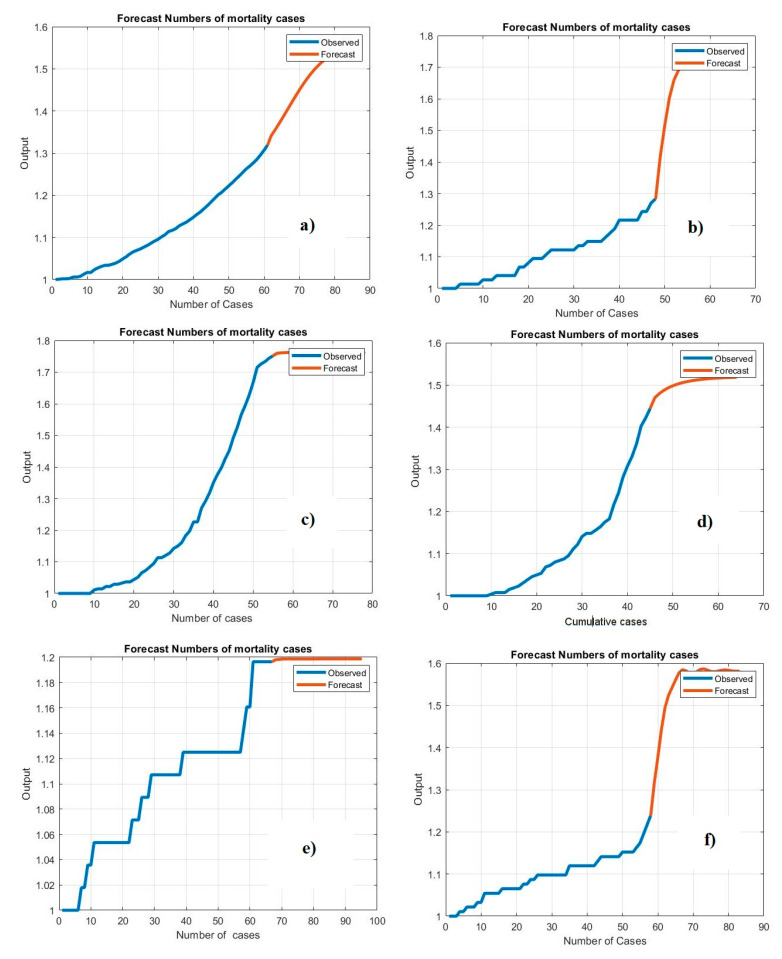
Performance of the Bi-LSTM model for predicting the number of confirmed cases using testing data: (**a**) Saudi Arabia, (**b**) Oman, (**c**) United Arab Emirates, (**d**) Kuwait, (**e**) Bahrain, and (**f**) Qatar.

Figure 8 shows the performance of the proposed system for forecasting the confirmed cases of COVID-19. The graphic representation shows the forecasting of unseen data. While the target (*x*-axis) values represent the numbers of simple confirmed cases, the output (*y*-axis) values represent the forecasting values.

**Figure 9:**
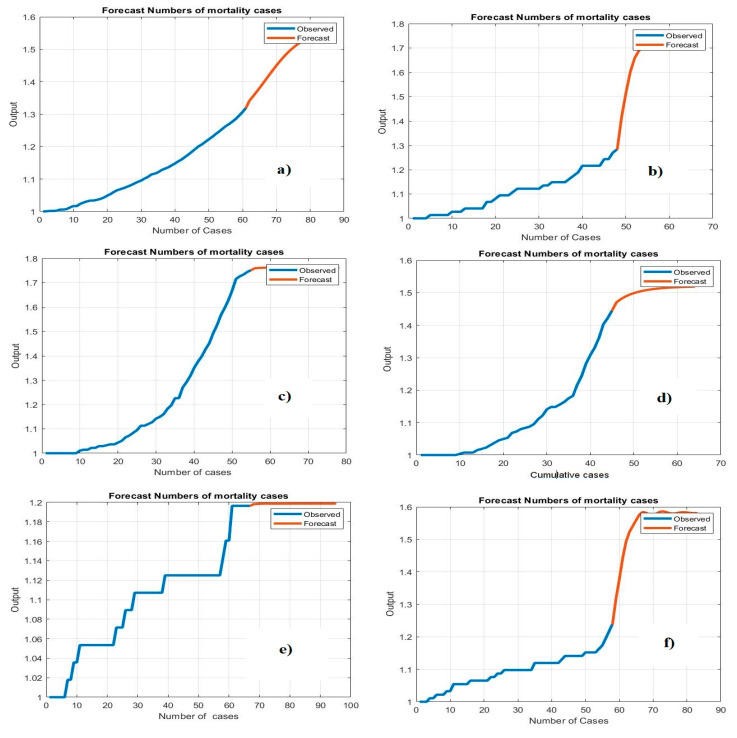
Prediction performance of the Bi-LSTM model in forecasting mortality cases using testing data: (**a**) Saudi Arabia, (**b**) Oman, (**c**) United Arab Emirates, (**d**) Kuwait, (**e**) Bahrain, and (**f**) Qatar.

Figure 9 shows the performance of the proposed system in forecasting the mortality cases of COVID-19. The graphic is a representation of the prediction of unseen data. While the target (*x*-axis) values represent numbers of simple confirmed cases, the output (*y*-axis) values represent the forecasting values.

Table 10 summarizes the prediction and observation values for the mortality cases of COVID-19 in the Gulf countries. The proposed system was evaluated and examined by taking 20% of the data for use in testing. Note that the mean errors of the prediction values are very close to the observation values.

## 5. Discussion

The COVID-19 pandemic has had a significant psychological and economic impact on the Gulf countries. At the end of January 2020, the Gulf Cooperation Council (GCC) declared the first case in the UAE [56]. Authorities in the GCC were quick to take high-level measures to contain the disease and prevent its spread; they did so much earlier than in other nearby countries [57]. The subsequent infections were therefore not as high in the UAE when compared with the massive spread that occurred in neighboring countries. Although the disease came from other countries, the lockdown significantly reduced the number of infections. All COVID-19-positive patients were placed in quarantine so that no one else would be infected, and the Ministry of Health in the Gulf states announced with each new infection that the person had been quarantined and those who had been in contact with positive cases were put under observation. The governments of Kuwait and Qatar quickly evacuated citizens from Iran, where the disease was spreading rapidly (the first case was announced on 19 February 2020), and then implemented strict decisions to suspend flights connecting to specific countries and advised citizens not to travel abroad [58]. By the end of May 2020, the number of active cases had increased in the Gulf countries, especially in Saudi Arabia, to reach 150,000 active cases.

We collected data from a public repository containing a record of COVID-19 cases around the world. For this study, we took a time interval from the first case that had appeared in Gulf countries until 08 June 2020. When COVID-19 appeared in the Gulf countries, only a few cases were reported because the Gulf countries imposed restrictions and measures to curb the spread of the epidemic. Modeling and prediction became important for estimating the number of active cases and deaths in the Gulf countries. Here, we proposed an advanced deep learning algorithm for predicting COVID-19 cases and attempted to assess the empirical evidence to predict the numbers of active cases and deaths in the Gulf countries. The Bi-LSTM model was applied to predict the cases, and a dataset was collected to test the proposed model. The dataset was divided into 80% training data and 20% testing data for evaluating the Bi-LSTM model. Table 11 shows the correlation coefficient results of the proposed system for predicting COVID-19 in the Gulf countries. Note that the Bi-LSTM model provided a correlation coefficient for predicting the active cases and mortality rate in the Gulf countries.

**Figure 10:**
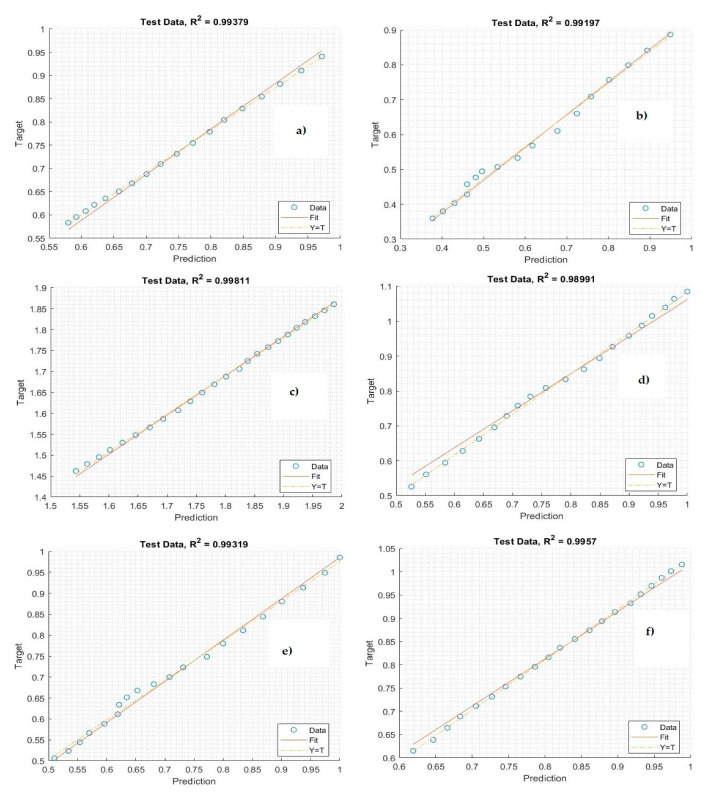
Plot diagrams for the prediction of active COVID-19 cases: (**a**) Saudi Arabia, (**b**) Oman, (**c**) United Arab Emirates, (**d**) Kuwait, (**e**) Bahrain, and (**f**) Qatar.

**Figure 11:**
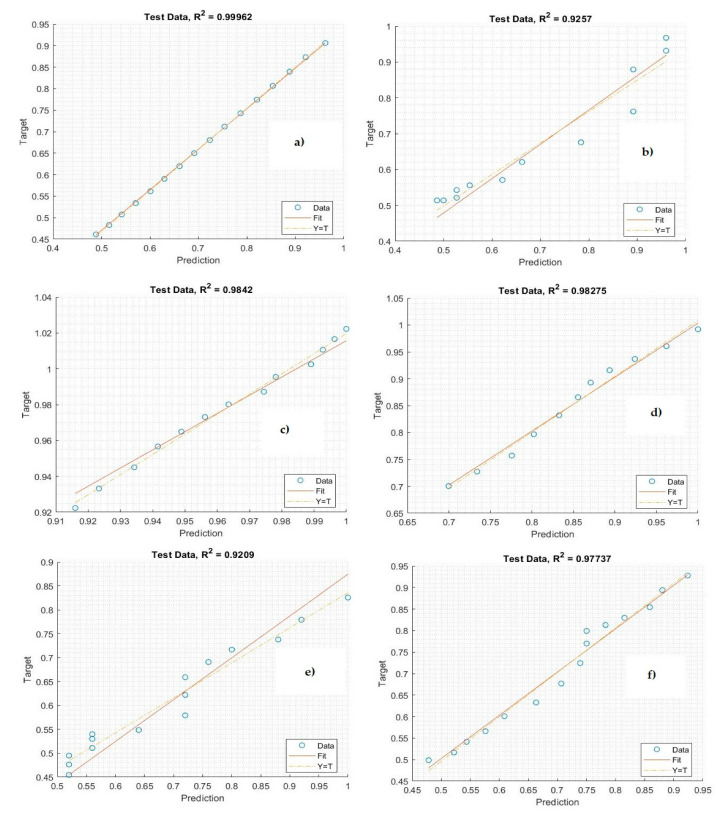
Plot Diagrams for the prediction mortality rate of COVID-19: (**a**) Saudi Arabia, (**b**) Oman, (**c**) United Arab Emirates, (**d**) Kuwait, (**e**) Bahrain, and (**f**) Qatar.

Figure 10 shows the results of the Bi-LSTM model for predicting active cases using the correlation coefficient. Notably, the regression plot is close to both the real and testing values. The values of the correlation coefficient metric are high for all countries, indicating that the Bi-LSTM model has a robust connection with the observation data. The correlation coefficient reached 99% between the actual data and prediction data. Figure 11 illustrates the regression plot of the Bi-LSTM model to predict mortality rates in the Gulf countries. Most of the correlation coefficient values are very high because of the relationship between the observed data and real data and the prediction of COVID-19 cases. These plots were employed to find the relationship between the predicted and the observation values by using Pearson’s correlation. While the *target* (*x*-axis) values represent the observation data, the *output* (*y*-axis) values represent the predicted values.

Modeling and prediction of the COVID-19 have played a pivotal and significant role in saving people’s lives. The deep learning algorithms were explored as an alternative approach to predict the numbers of confirmed and death cases of COVID-19. Table 12 summarizes existing system results against the proposed system results. Numerous studies have employed artificial intelligence models for estimating and predicting COVID-19.

Stevenson et al. [81] applied three models (LSTM, naïve, and seasonal naïve forecast), to predict COVID-19 and they used RMSE metrics to evaluate the obtained results for 7-day and 14-day time intervals to predict future daily cases in South Africa. Satu et al. [82] proposed polynomial multi-layer perceptron (Poly-MLP), support vector regression (SVR), and Prophet models to predict confirmed and death cases using two evaluation metrics, RMSE and R2, to examine the proposed models. The datasets were collected using the Bangladesh surveillance system for the time period of 185 days from 8 March 2020 to 28 November 2020 to predict confirmed infection, death, and recovered cases. However, the authors still required 34 iterative rounds to reach these results, and they had to change neuron values in each round.

Zisad et al. [83] used SEIR and SIRNN models to predict COVID-19. The model was tested and trained to employ available data from 250 days in Bangladesh. The RMSE, NRMSE, and R2 were used to evaluate the proposed system, and the prediction error decreased from NRMSE = 1.99 to NRMSE = 0.68, while the accuracy of the regression metric of the proposed system for confirmed cases was between 90% and 99%.

Awwad et al. [84] used the statistical STARMA model to predict confirmed and death cases in Saudi Arabian cities. This study covered most Saudi Arabian cities in the time interval from 23 March 2020 to 28 May 2020. The proposed system was tested using the RMSE metric, and we selected the superior result obtained by their proposed model for RMSE = 0.339 for predicting confirmed COVID-19 cases in Taif and RMSE = 0.368 for predicting confirmed cases in Jeddah.

Tandon et al. [85] presented an ARIMA model to predict future COVID-19 infection cases using datasets collected from 18 March 2020 or 22 January 2020 to 13 April 2020 round (18 days) from different countries (the USA, China, and Italy) to examine the proposed system. The MAPE metrics were used to test the results of the system, with the best accuracy of the model achieved as MAPE = 4.1.

In the current research, we used the Bi-LSTM model for modeling and predicting COVID-19 in the Gulf countries, and achieved excellent results, with RMSE = 0.00019, for predicting confirmed cases at the training phase. For predicting death cases, the proposed system gave RMSE = 0.00014 and very low prediction errors, with correlation regression values of 99.98 to94.16 and 99.83 to 94.16% for prediction of confirmed and death cases, respectively, in the training phase. We compared the results of the proposed model with existing systems that used the same metrics, and we confirmed that the proposed system achieved superior results when compared with other selective studies related to COVID-19 predictions. Overall, the proposed system is better than the existing systems.

## 6. Conclusions

Over the past year, Gulf countries have imposed restrictions and measures to curb the spread of the COVID-19 epidemic, putting responsible officers in charge of millions of dollars to accomplish this goal. The development and modeling of accurate prediction models are vital steps for estimating the COVID-19 outbreak to provide insights into the spread of this infectious disease. This paper presented the Bi-LSTM network model to predict COVID-19 in the Gulf countries to control the spread of the COVID-19 infectious disease. Initially, real data were collected from a publicly available website that contains up-to-date records of numbers of COVID-19 patients. The results of the Bi-LSTM network showed a high generalization capability for predicting the number of confirmed cases and deaths in the Gulf countries according to the statically analyzed metrics. The dataset was divided into a training set and a testing set. The testing values were used to validate the performance of the Bi-LSTM model. According to the obtained results, the proposed model showed high prediction for most of the Gulf countries’ data. The Bi-LSTM model demonstrated that promising results can be gained with fewer errors using this approach. Future studies using another deep learning algorithm are strongly encouraged to improve the results of the existing model.

## Figures and Tables

**Table 1:** Some of the control measures taken by Gulf countries faced with COVID-19 in 2020.

Date	Countries	Responses
6 February 2020	Saudi Arabia	Suspension of flights from and to China
27 February 2020 28 February 2020		Suspension of Umrah and tourism for foreign nationals
5 March 2020		Prevention of congregational prayers in Makkah mosques
8 March 2020		Imposition of lockdown in Al-Qatif
9 March 2020		Closure of schools and universities
14 March 2020		Suspension of flights to the European Union countries
17 March 2020		Canceling of sports events and closure of mosques
25 March 2020		Movement between provinces banned; lockdown imposed in Makkah, Madinah, and Riyadh; leaving homes prohibited from 3 pm to 6 am
29 March 2020		Lockdown imposed in Jeddah; people allowed to buy essentials only from 6 am to 3 pm
31 January 2020	UAE	Care and treatment for COVID-19 patients provided free of charge
28 February 2020		Imposition of home quarantine for people in contact with COVID-19 patients
		Shutdown of some hotels
8 March 2020		Closure of schools and universities
15 March 2020		Shutdown of cinemas, gyms, and parks
22 March 2020		Prevention of COVID-19 spread through disinfection and sanitization campaigns
25 March 2020		Banning travel to and from the country.
26 March 2020		Imposition of night curfew
28 March 2020		Launch of first drive-through COVID-19 testing sites
4 April 2020		Imposition of 24 h curfew in Dubai
10 April 2020		Addition of 13 drive-through testing centers for COVID-19
12 April 2020		Operation of repatriation flights
12 March 2020	Kuwait	Shutdown of schools and universities
12 March, 2020		Imposition of lockdown till 26 March 2020
13 March 2020		Cessation of operation of international flights
26 March 2020		Shutdown of some shops, restaurants, and mosques
9 March 2020	Oman	Cessation of operations from and to Milan and Italy
12 March 2020		Suspension of flights from and to Saudi Arabia
		Issuance of orders to form a supreme committee to deal with COVID-19
14 March 2020		Shutdown of schools and universities
15 March 2020		Cessation of visa issuance to all countries, banning of sports events
17 March 2020		Banning of congregational prayers in mosques and large gatherings
18 March 2020		Suspension of entry into the country, even for people from GCC countries
22 March 2020		Banning of more flights from different countries
27 March 2020		Prevention of COVID-19 through disinfection and sanitization campaigns
29 March 2020		Banning of nationwide and international flights
12 April 2020		Prevention of movements between governorates
20 April 2020		Extension of the lockdown in Muscat till 8 May 2020. Prevention of social gatherings and sports events in Ramadan
28 April 2020		Reopening of some businesses and commercial establishments
4 May 2020		Extension of the lockdown in Muscat till 29 May 2020
24 February 2020	Bahrain	Banning of flights from and to Iran and the UAE
25 February 2020		Shutdown of schools and universities
26 February 2020		Banning of flights from and to Iraq and Lebanon
26 February 2020		Medical examinations ordered for travelers from Iran
23 March 2020		Shutdown of mosques.
24 March 2020		Limited time given for people to buy essentials from 5 am to 6 pm
		Prevention of disinfectant exports
26 March 2020		Closure and regulation of some shops and restaurants
30 March 2020		Launch of COVID-19 testing for the people
31 March 2020		Release of a mobile application to alert people of COVID-19 patients and the areas they have visited
22 April 2020		Prevention of social gatherings and sports throughout Ramadan
14 May 2020		Private hospitals granted permission to start COVID-19 testing
9 March 2020	Qatar	Shutdown of schools and universities Suspension of international flights from and to the countries hit by COVID-19
14 March 2020		Suspension of more international flights
16 March 2020		Prevention of congregational prayers in mosques
21 March 2020		Shutdown of all cafes, restaurants, and beaches

**Table 2:** Numbers of confirmed cases of COVID-19 in the Gulf countries.

Country	Start Date	Number of Simples	Time Period	End Date
Saudi Arabia	3 March 2020	93	146 days	8 June 2020
Oman	25 February 2020	86	164 days	
Kuwait	24 February 2020	124	165 days	
Bahrain	24 February 2020	100	164 days	
United Arab Emirates	27 January 2020	103	192 days	
Qatar	1 March 2020	106	281 days	

**Table 3:** Number of deaths from COVID-19 in the Gulf countries.

Countries	Start Date	Number of Simples	Time Periods	End Date
Saudi Arabia	25 March 2020	85	135 days	8 June 2020
Oman	1 April 2020	64	280 days	
Kuwait	5 April 2020	69	124 days	
Bahrain	17 March 2020	55	144 days	
United Arab Emirates	22 March 2020	84	137 days	
Qatar	29 March 2020	80	130 days	

**Table 4:** Significant parameter values of the Bi-LSTM model.

Parameters of the LSTM Algorithm	
Number of hidden layers	200
Number of delays	[1–3]
Number of shallow hidden layers	[3050]
Number Max epochs used in model	400
Number of Mini batch size used in model	64
Execute environment	CPU
Number of Max iterations	250
Dropout	0.5
Optimization function	Adam

**Table 5:** Results of Bi-LSTM model to predict the number of confirmed cases during COVID-19 at the training stage.

Metrics	Saudi Arabia	Oman	UAE	Kuwait	Bahrain	Qatar
MSE	4.4217 × 10^−5^	03.9035 × 10^−5^	0.000916	2.1228 × 10^−5^	5.7006 × 10^−5^	2.1719 × 10^−5^
RMSE	0.00664	0.0062	0.030	0.00460	0.00755	0.00466
NRMSE	0.00372	0.0633	0.077	0.0528	0.06109	0.032

**Table 6:** Results of the Bi-LSTM model for predicting the number of deaths cases during COVID-19 at the training stage.

	Saudi Arabia	Oman	UAE	Kuwait	Bahrain	Qatar
MSE	3.8242 × 10^−5^	0.000169	0.000466	0.00197	0.000537	0.00134
RMSE	0.00618	0.013025	0.02159	0.0140	0.02317	0.0116
NRMSE	0.036	0.0763	0.0530	0.0582	0.0894	0.0777

**Table 7:** Testing results of the Bi-LSTM model to predict the number of confirmed cases.

	Saudi Arabia	Oman	UAE	Kuwait	Bahrain	Qatar
MSE	0.00014	0.00161	0.0116	0.00149	0.000111	0.0005095
RMSE	0.01222	0.0401	0.1081	0.0386	0.01053	0.02257
NRMSE	0.0163	0.0651	0.0607	0.0509	0.01453	0.02761

**Table 8:** Testing results of the Bi-LSTM model to predict the number of mortality cases during the COVID-19 pandemic in the Gulf countries.

	Saudi Arabia	Oman	UAE	Kuwait	Bahrain	Qatar
MSE	0.00140	0.00354	0.000205	0.000152	0.00780	0.000431
RMSE	0.0374	0.0595	0.0143	0.01233	0.08835	0.020779
NRMSE	0.052	0.0853	0.0148	0.0145	0.1274	0.02941

**Table 9:** Comparison between target values and prediction values at the testing stage for predicting confirmed cases.

Oman			Saudi Arabia		
Errors	Prediction	Target	Errors	Prediction	Target
0.0162	0.361	0.3772	0.0079	0.5877	0.5798
0.02	0.3815	0.4023	0.0078	0.6	0.5922
0.024	0.4048	0.4297	0.0062	0.6129	0.6066
0.03	0.4301	0.4601	0.0066	0.6265	0.6198
0.001	0.4591	0.4601	0.0031	0.64027	0.637
0.002	0.4785	0.4808	0.0021	0.6556	0.6578
0.00021	0.4961	0.4959	0.0048	0.6732	0.6781
0.024	0.5088	0.5335	0.0073	0.6932	0.7005
0.046	0.5347	0.5816	0.0074	0.71492	0.7223
0.046	0.5704	0.6173	0.0101	0.7369	0.7471
0.065	0.6123	0.6774	0.0114	0.7605	0.7719
0.061	0.6622	0.7239	0.0131	0.7848	0.798
0.046	0.7114	0.7581	0.0098	0.8105	0.8204
0.042	0.759	0.8018	0.0131	0.8354	0.8486
0.046	0.8013	0.8479	0.0172	0.8613	0.8786
0.049	0.8437	0.8936	0.0184	0.8885	0.907
0.059	0.8896	0.9486	0.022	0.9177	0.9397
			0.023	0.9482	0.9713
**Kuwait**			**UAE**		
**Errors**	**Prediction**	**Target**	**Errors**	**Prediction**	**Target**
0.0012	0.525	0.5263	0.0797	1.4635	1.5432
0.00798	0.5595	0.5515	0.0823	1.4802	1.5625
0.00687	0.5911	0.5842	0.0863	1.4967	1.583
0.01071	0.6249	0.6142	0.088	1.5138	1.6018
0.01504	0.6575	0.6425	0.0922	1.531	1.6233
0.022	0.6908	0.6688	0.0967	1.549	1.6458
0.0321	0.7218	0.6897	0.1023	1.5677	1.67
0.0404	0.7492	0.7088	0.1051	1.5879	1.693
0.0427	0.7732	0.7305	0.11	1.6086	1.7187
0.0387	0.7958	0.757	0.1093	1.6302	1.7396
0.0298	0.8205	0.7907	0.1089	1.6507	1.7597
0.0283	0.8506	0.8223	0.1104	1.6704	1.7809
0.035	0.8841	0.8491	0.112	1.6889	1.801
0.0454	0.9171	0.8716	0.1166	1.7071	1.8237
0.0464	0.946	0.8995	0.112	1.7262	1.8382
0.0518	0.9737	0.9218	0.1116	1.743	1.8547
0.0602	0.9997	0.9394	0.1144	1.7589	1.8734
0.061	1.0236	0.9621	0.1166	1.7738	1.8904
0.068	1.0458	0.97748	0.1174	1.7893	1.9068
			0.1173	1.8047	1.9221
			0.1176	1.8192	1.9368
			0.1208	1.833	1.9538
			0.1228	1.8471	1.9699
			0.1247	1.8613	1.986
**Qatar**			**Bahrain**		
**Errors**	**Prediction**	**Target**	**Errors**	**Prediction**	**Target**
0.0017	0.6208	0.6191	0.00067	0.5095	0.5102
0.0021	0.6444	0.6466			
0.004	0.6706	0.6659			
0.0115	0.6953	0.6837	0.00573	0.5285	0.5343
0.0128	0.7179	0.7051	0.00257	0.5511	0.5537
0.0111	0.7385	0.7273	0.0048	0.5748	0.5699
0.0142	0.7601	0.7458	0.00043	0.5958	0.5962
0.0155	0.7819	0.7664	0.00116	0.6178	0.619
0.017	0.8033	0.7863	0.0197	0.6409	0.6212
0.0189	0.8239	0.8049	0.0241	0.6586	0.6344
0.0235	0.8445	0.8209	0.0194	0.6719	0.6525
0.0223	0.8634	0.8411	0.0031	0.684	0.6809
0.0213	0.8825	0.8611	0.0024	0.7053	0.7078
0.0236	0.902	0.8784	0.0001	0.7312	0.7311
0.0257	0.9219	0.8962	0.0134	0.7586	0.772
0.0233	0.9409	0.9176	0.0087	0.7908	0.7996
0.0289	0.9604	0.9314	0.01104	0.8229	0.8339
0.0323	0.9786	0.9463	0.0094	0.8585	0.868
0.0354	0.9958	0.9604	0.0086	0.892	0.9006
0.0372	1.0105	0.9732	0.0102	0.9269	0.9371
0.0367	1.0248	0.9881	0.0117	0.9625	0.9743

**Table 10:** Comparison between target values and prediction values at the testing stage for predicting mortality cases.

Oman			Saudi Arabia		
Errors	Prediction	Target	Errors	Prediction	Target
0.0394	0.4864	0.5258	0.0243	0.4643	0.4886
0.0302	0.5	0.5302	0.0294	0.4862	0.5156
0.0085	0.527	0.5356	0.0308	0.511	0.5418
0.024	0.527	0.551	0.0328	0.5378	0.5706
0.01	0.554	0.5649	0.0353	0.5657	0.601
0.037	0.6216	0.5844	0.035	0.5947	0.6298
0.041	0.66216	0.6206	0.0365	0.6245	0.661
0.114	0.78378	0.669	0.0364	0.6551	0.6915
0.139	0.89189	0.7526	0.0379	0.6856	0.7236
0.041	0.89189	0.85	0.0367	0.7172	0.754
0.033	0.9594	0.9258	0.0384	0.7485	0.787
0.0265	0.9594	0.9859	0.0402	0.7806	0.8208
			0.0405	0.8132	0.8538
			0.0419	0.8465	0.8884
			0.0411	0.8803	0.9214
			0.0482	0.9137	0.962
**Kuwait**			**UAE**		
**Errors**	**Prediction**	**Target**	**Errors**	**Prediction**	**Target**
0.0005	0.6991	0.6996		0.9212	0.9161
0.0079	0.7259	0.7338		0.9321	0.9234
0.0204	0.7553	0.7757		0.9437	0.9343
0.0074	0.7948	0.8023		0.9554	0.9416
0.0027	0.8299	0.8327		0.9636	0.9489
0.008	0.8637	0.8555		0.9718	0.9562
0.02	0.8909	0.8707		0.9788	0.9635
0.0204	0.914	0.8935		0.9859	0.9745
0.01	0.9348	0.924		0.9942	0.9781
0.0035	0.9584	0.962		1.001	0.9891
				1.0093	0.9927
				1.0151	0.9964
**Qatar**			**Bahrain**		
**Errors**	**Prediction**	**Target**	**Errors**	**Prediction**	**Target**
0.018	0.4963	0.4782	0.0642	0.4557	0.52
0.0076	0.5141	0.5217	0.0376	0.4823	0.52
0.0045	0.539	0.5434	0.014	0.5059	0.52
0.0126	0.5634	0.576	0.0429	0.517	0.56
0.0106	0.5981	0.6086	0.0288	0.5311	0.56
0.0332	0.6298	0.663	0.0164	0.5436	0.56
0.0326	0.6738	0.7065	0.0853	0.5547	0.64
0.0182	0.7209	0.7391	0.1372	0.5828	0.72
0.0161	0.7662	0.75	0.0843	0.6356	0.72
0.0453	0.7953	0.75	0.0375	0.6825	0.72
0.0264	0.809	0.7826	0.0554	0.7045	0.76
0.0104	0.8257	0.8152	0.0815	0.7184	0.8
0.0083	0.8504	0.8586	0.1353	0.7447	0.88
0.0088	0.8893	0.8804	0.1243	0.7957	0.92
0.00086	0.9231	0.9239			

**Table 11:** Prediction results of the Bi-LSTM model using the R^2^ metric at the testing phase.

Number of Confirmed Cases			Number of Mortality Cases	
	Training (%)	Testing (%)	Training (%)	Testing (%)
Saudi Arabia	98.988	99.37	99.83	99.96
Oman	99.70	99.19	99.01	92.57
UAE	94.16	99.81	99.53	98.42
Kuwait	99.91	98.99	99.52	98.27
Bahrain	99.77	99.31	94.16	92.09
Qatar	99.94	99.57	98.88	97.73

**Table 12:** Comparison of the proposed system with existing models.

Author/References	Models	Region	Time Periods	RMSE	R^2^	NRMSE
Stevenson et al. [81]	LSTM RNN model Naïve Seasonal Naïve Forecast	South Africa	14 and 7 days	76.57 89.43 79.99		
Satu et al. [82]	Poly-MLP	Bangladesh	8 march 2020 to 28 November 2020	2.3675	93.94	
	SVR			2.4589	76.60	
	Prophet			1.3350	98.77	
Zisad et al. [83]	SEIR model SIR with NN	Bangladesh	250 days	0.797 0.835	0.917	1.99 to 0.68
Awwad et al. [84]	STARM	Saudi Arabia	23 March 2020 to 28 May 2020	0.339 (Taif city) 0.368 (Jeddah (city))		
Tandon et al. [85]	ARIMA	USA, Chain, Italy France, others	18 days 22 January 2020 to 13 April 2020		4.1	
Proposed system	Bi-LSTM ( predict confirmed cases )	Saudi Arabia, Oman, the United Arab Emirates (UAE), Kuwait, Bahrain, and Qatar	Maximum 280 days Minimum 124 days	0.00019 (Training)	99.98–94.16	0.016
	Bi-LSTM (predict death cases)	Saudi Arabia, Oman, the United Arab Emirates (UAE), Kuwait, Bahrain, and Qatar		0.00014(Training)	99.83–94.16	0.077

## Data Availability

The data presented in this study are available here https://ourworldindata.org/coronavirus-testing (accessed on 9 October 2021).

## References

[1] Rismanbaf A. (2020). Potential Treatments for COVID-19; A Narrative Literature Review. Arch. Acad. Emerg. Med..

[2] (2020). Coronavirus Disease (COVID-19) Pandemic. https://www.who.int/emergencies/diseases/novel-coronavirus-2019.

[3] Velavan T.P., Meyer C.G. (2020). The COVID-19 epidemic. Trop. Med. Int. Health.

[4] Letko M., Marzi A., Munster V. (2020). Functional Assessment of Cell Entry and Receptor Usage for SARS-CoV-2 and Other Lineage B Betacoronaviruses. Nat. Microbiol..

[5] World Health Organization (2020). Coronavirus Disease 2019 (COVID-19) Situation Report–29.

[6] Elsevier (2020). Novel Coronavirus Information Center. https://www.elsevier.com/connect/coronavirus-information-center.

[7] World Health Organization (2020). Report of the WHO-China Joint Mission on Coronavirus Disease 2019 (COVID-19).

[8] Hui D.S., Azhar E.I., Madani T.A., Ntoumi F., Kock R., Dar O., Ippolito G., Mchugh T.D., Memish Z.A., Drosten C. (2020). The Continuing 2019-Ncov Epidemic Threat of Novel Coronaviruses to Global Health the Latest 2019 Novel Coronavirus Outbreak in Wuhan, China. Int. J. Infect. Dis..

[9] Remuzzi A., Remuzzi G. (2020). COVID-19 and Italy: What next?. Lancet.

[10] Ivanov D. (2020). Predicting the Impacts of Epidemic Outbreaks on Global Supply Chains: A Simulation-Based Analysis on the Coronavirus Outbreak (COVID-19/SARS-CoV-2) Case. Transp. Res. Part E Logist. Transp. Rev..

[11] Koolhof I.S., Gibney K.B., Bettiol S., Charleston M., Wiethoelter A., Arnold A.L., Campbell P.T., Neville P.J., Aung P., Shiga T. (2020). The Forecasting of Dynamical Ross River Virus Outbreaks: Victoria, Australia. Epidemics.

[12] Darwish A., Rahhal Y., Jafar A. (2020). A Comparative Study on Predicting Influenza Outbreaks Using Different Feature Spaces: Application of Influenza-Like Illness Data from Early Warning Alert and Response System in Syria. BMC Res. Notes.

[13] Rypdal M., Sugihara G. (2019). Inter-Outbreak Stability Reflects the Size of the Susceptible Pool and Forecasts Magnitudes of Seasonal Epidemics. Nat. Commun..

[14] Scarpino S.V., Petri G. (2019). On the Predictability of Infectious Disease Outbreaks. Nat. Commun..

[15] Zhan Z., Dong W., Lu Y., Yang P., Wang Q., Jia P. (2019). Real-Time Forecasting of Hand-Foot-and-Mouth Disease Outbreaks Using the Integrating Compartment Model and Assimilation Filtering. Sci. Rep..

[16] Lixiang L., Yang Z., Dang Z., Meng C., Huang J., Meng H., Wang D., Chen G., Zhang J., Peng H. (2020). Propagation analysis and prediction of the COVID-19. Infect. Dis. Model..

[17] Ciufolini I., Paolozzi A. (2020). Mathematical Prediction of the Time Evolution of the COVID-19 Pandemic in Italy by a Gauss Error Function and Monte Carlo Simulations. Eur. Phys. J. Plus.

[18] Pham H. (2020). On Estimating the Number of Deaths Related to Covid-19. Mathematics.

[19] Cakir Z., Savas H.B. (2020). A Mathematical Modelling Approach in the Spread of the Novel 2019 Coronavirus SARS-CoV-2 (COVID-19) Pandemic. Electr. J. Gen. Med..

[20] Alimadadi A., Aryal S., Manandhar I., Munroe P.B., Joe B., Cheng X. (2020). Artificial Intelligence and Machine Learning to Fight COVID-19. Physiol. Genom..

[21] Tarnok A. (2020). Machine Learning, COVID-19 (2019-nCoV), and Multi-OMICS. Cytom. A.

[22] Wynants L., Van Calster B., Bonten M.M.J., Collins G.S., Debray T.P.A., De Vos M., Haller M.C., Heinze G., Moons K.G.M., Riley R.D. (2020). Prediction Models for Diagnosis and Prognosis of Covid-19 Infection: Systematic Review and Critical Appraisal. Brit. Medic. J..

[23] Singh D., Kumar V., Kaur M. (2020). Classification of COVID-19 Patients from Chest CT Images Using Multi-Objective Differential Evolution-Based Convolutional Neural Networks. Eur. J. Clin. Microbiol. Infect. Dis..

[24] Naude W. (2020). Artificial intelligence vs COVID-19: Limitations, Constraints and Pitfalls. AI Soc..

[25] Kim S., Seo Y.B., Jung E. (2020). Prediction of COVID-19 Transmission Dynamics Using a Mathematical Model Considering Behavior Changes in Korea. Epidem. Health.

[26] Zhu Y.F., Chen Y.Q. (2020). On a Statistical Transmission Model in Analysis of the Early Phase of COVID-19 Outbreak. Statist. Biosci..

[27] Bai Z.H., Gong Y., Tian X.D., Cao Y., Liu W.J., Li J. (2020). The Rapid Assessment and Early Warning Models for COVID-19. Virol. Sin..

[28] Wolfram C. (2020). An Agent-Based Model of Covid-19. Complex Syst..

[29] (2020). National Center for Immunization and Respiratory Diseases (NCIRD). Covid-19 Forecasts. https://www.cdc.gov/coronavirus/2019-ncov/covid-data/forecasting-us.html.

[30] Sperrin M., Grant S.W., Peek N. (2020). Prediction Models for Diagnosis and Prognosis in Covid-19. Brit. Medic. J..

[31] Shi H., Han X., Zheng C. (2020). Evolution of CT Manifestations in a Patient Recovered from 2019 Novel Coronavirus (2019-Ncov) Pneumonia in Wuhan, China. Radiology.

[32] Xu X., Chen P., Wang J., Feng J., Zhou H., Li X., Zhong W., Hao P. (2020). Evolution of the Novel Coronavirus from the Ongoing Wuhan Outbreak and Modeling of Its Spike Protein for Risk of Human Transmission. Sci. China Life Sci..

[33] Lau S.K., Lee P., Tsang A.K., Yip C.C., Tse H., Lee R.A., So L.Y., Lau Y.L., Chan K.H., Woo P.C. (2011). Molecular Epidemiology of Human Coronavirus OC43 Reveals Evolution of Di_Erent Genotypes over Time and Recent Emergence of a Novel Genotype Due to Natural Recombination. J. Virol..

[34] Seo D.W., Shin S.Y. (2017). Methods Using Social Media and Search Queries to Predict Infectious Disease Outbreaks. Healthc. Inform. Res..

[35] Meyers L.A., Pourbohloul B., Newman M.E., Skowronski D.M., Brunham R.C. (2005). Network Theory and SARS: Predicting Outbreak Diversity. J. Theory Biol..

[36] Alessa A., Faezipour M. (2018). A Review of Influenza Detection and Prediction Through Social Networking Sites. Theor. Biol. Med. Model..

[37] Liu D., Guo S., Zou M., Chen C., Deng F., Xie Z., Hu S., Wu L. (2019). A Dengue Fever Predicting Model Based on Baidu Search Index Data and Climate Data in South China. PLoS ONE.

[38] Shin S., Seo D., An J., Kwak H., Kim S., Gwack J., Jo M. (2016). High Correlation of Middle East Respiratory Syndrome Spread with Google Search and Twitter Trends in Korea. Sci. Rep..

[39] Xu Q., Gel Y.R., Ramirez Ramirez L.L., Nezafati K., Zhang Q., Tsui K.L. (2017). Forecasting Influenza in Hong Kong with Google Search Queries and Statistical Model Fusion. PLoS ONE.

[40] He F., Hu Z., Zhang W., Cai L., Cai G., Aoyagi K. (2017). Construction and Evaluation of Two Computational Models for Predicting the Incidence of Influenza in Nagasaki Prefecture, Japan. Sci. Rep..

[41] Najafabadi M.M., Villanustre F., Khoshgoftaar T.M., Seliya N., Wald R., Muharemagic E. (2017). Deep Learning Applications and Challenges in Big Data Analytics. J. Big Data.

[42] Janowczyk A., Madabhushi A. (2016). Deep Learning for Digital Pathology Image Analysis: A Comprehensive Tutorial with Selected Use Cases. J. Pathol. Inform..

[43] Esteva A., Kuprel B., Novoa R.A., Ko J., Swetter S.M., Blau H.M., Thrun S. (2017). Dermatologist-Level Classification of Skin Cancer with Deep Neural Networks. Nature.

[44] Bychkov D., Linder N., Turkki R., Nordling S., Kovanen P.E., Verrill C., Walliander M., Lundin M., Haglund C., Lundin J. (2018). Deep Learning Based Tissue Analysis Predicts Outcome in Colorectal Cancer. Sci. Rep..

[45] Aldhyani T.H., Alshebami A.S.A., Alzahrani M.Y. (2020). Soft Computing Model to Predict Chronic Diseases. J. Inf. Sci. Eng..

[46] Aldhyani T.H., Alshebami A.S., Alzahrani M.Y. (2020). Soft Clustering for Enhancing the Diagnosis of Chronic Diseases over Machine Learning Algorithms. J. Healthc. Eng..

[47] Balcan D., Colizza V., Goncalves B., Hu H., Ramasco J., Vespignani A. (2009). Multiscale Mobility Networks And The Spatial Spreading Of Infectious Diseases. Proc. Natl. Acad. Sci. USA.

[48] Yuan Q., Nsoesie E., Lv B., Peng G., Chunara R., Brownstein J. (2013). Monitoring Influenza Epidemics in China with Search Query from Baidu. PLoS ONE.

[49] Milinovich G.J., Avril S.M., Clements A.C., Brownstein J.S., Tong S., Hu W. (2014). Using Internet Search Queries for Infectious Disease Surveillance: Screening Diseases for Suitability. BMC Infect. Dis..

[50] Cook S., Conrad C., Fowlkes A., Mohebbi M. (2011). Assessing Google Flu Trends Performance in the United States during the 2009 Influenza Virus A (H1N1) Pandemic. PLoS ONE.

[51] Zain Z.M., Alturki N.M. (2021). COVID-19 Pandemic Forecasting Using CNN-LSTM: A Hybrid Approach. J. Control. Sci. Eng..

[52] Vynnycky E., White R. (2010). An Introduction to Infectious Disease Modelling.

[53] Aldhyani T.H., Alrasheed M., Alzahrani M.Y., Ahmed H. (2020). Deep Learning and Holt-Trend Algorithms for Predicting COVID-19 Pandemic. medRxiv.

[54] Lawson A.B. (2016). Statistical Methods in Spatial Epidemiology.

[55] Alotaibi A., Shiblee M., Alshahrani A. (2021). Prediction of Severity of COVID-19-Infected Patients Using Machine Learning Techniques. Computers.

[56] MOHAP-UAE, Ministry of Health and Prevention (MOHAP), United Arab of Emirates. SEHA Opens 13 Additional Drive-through COVID-19 Testing Centres. https://www.mohap.gov.ae/en/MediaCenter/News/Pages/2365.aspx.

[57] Alandijany T.A., Faizo A.A., Azhar E.I. (2020). Coronavirus Disease of 2019 (COVID-19) in The Gulf Cooperation Council (GCC) Countries: Current Status and Management Practices. J. Infect. Public Health.

[58] MOPH-Qatar, Ministry of Public Health, Qatar. https://twitter.com/MOPHQatar/status/1233744372556029952?s=20.

[59] Shahid F., Zameer A., Muneeb M. (2020). Predictions For COVID-19 with Deep Learning Models of LSTM, GRU and Bi-LSTM. Chaos Solitons Fractals.

[60] Chakraborty C., Banerjee A., Garg L., Rodrigues J.J.P.C. (2020). Studies in Big Data.

[61] Rustam F., Reshi A.A., Mehmood A., Ullah S., On B., Aslam W., Choi G.S. (2020). COVID-19 Future Forecasting Using Supervised Machine Learning Models. IEEE Access.

[62] Ochodek M., Kopczyńska S., Staron M. (2020). Deep Learning Model for End-to-End Approximation of COSMIC Functional Size Based on Use-Case Names. Inf. Softw. Technol..

[63] Hu F., Zhu Y., Liu J., Li L. (2020). An Efficient Long Short-Term Memory Model Based on Laplacian Eigenmap in Artificial Neural Networks. Appl. Soft Comput..

[64] Wen S., Wang Y., Tang Y., Xu Y., Li P., Zhao T. (2019). Real-Time Identification of Power Fluctuations Based on LSTM Recurrent Neural Network: A Case Study on Singapore Power System. IEEE Trans. Ind. Inform..

[65] Musulin J., Baressi Šegota S., Štifanić D., Lorencin I., Anđelić N., Šušteršič T., Blagojević A., Filipović N., Ćabov T., Markova-Car E. (2021). Application of Artificial Intelligence-Based Regression Methods in the Problem of COVID-19 Spread Prediction: A Systematic Review. Int. J. Environ. Res. Public Health.

[66] Al-Turaiki I., Almutlaq F., Alrasheed H., Alballa N. (2021). Empirical Evaluation of Alternative Time-Series Models for COVID-19 Forecasting in Saudi Arabia. Int. J. Environ. Res. Public Health.

[67] Abuhasel K.A., Khadr M., Alquraish M.M. (2020). Analyzing and Forecasting COVID-19 Pandemic in the Kingdom of Saudi Arabia Using ARIMA and SIR Models. Comput. Intell..

[68] Omran N.F., Abd-el Ghany S.F., Saleh H., Ali A.A., Gumaei A., Al-Rakhami M. (2021). Applying Deep Learning Methods on Time-Series Data for Forecasting COVID-19 in Egypt, Kuwait, and Saudi Arabia. Complexity.

[69] Ardabili S.F., Mosavi A., Ghamisi P., Ferdinand F., Varkonyi-Koczy A.R., Reuter U., Rabczuk T., Atkinson P.M. (2020). COVID-19 Outbreak Prediction with Machine Learning. Algorithms.

[70] Pinter G., Felde I., Mosavi A., Ghamisi P., Gloaguen R. (2020). COVID-19 Pandemic Prediction for Hungary; A Hybrid Machine Learning Approach. Mathematics.

[71] Yuan J., Wang H., Lin C., Liu D., Yu D. (2019). A Novel GRU-RNN Network Model For Dynamic Path Planning of Mobile Robot. IEEE Access.

[72] Jamshidi M., Lalbakhsh A., Talla J., Peroutka Z., Hadjilooei F., Lalbakhsh P., Jamshidi M., La Spada L., Mirmozafari M., Dehghani M. (2020). Artificial Intelligence and COVID-19: Deep Learning Approaches for Diagnosis and Treatment. IEEE Access.

[73] Jamshidi M.B., Lalbakhsh A., Talla J., Peroutka Z., Roshani S., Matousek V., Roshani S., Mirmozafari M., Malek Z., La Spada L., Arpaci I., Al-Emran M., Al-Sharafi M.A., Marques G. (2021). Deep Learning Techniques and COVID-19 Drug Discovery: Fundamentals, State-of-the-Art and Future Directions. Emerging Technologies During the Era of COVID-19 Pandemic.

[74] Zhiheng H., Wei X., Yu K. (2015). Bidirectional LSTM-CRF Models for Sequence Tagging. arXiv.

[75] Kafieh R., Saeedizadeh N., Arian R., Amini Z., Serej N.D., Vaezi A., Javanmard S.H. (2020). Isfahan and COVID-19: Deep spatiotemporal representation. Chaos Solitons Fractals.

[76] Wang P., Zheng X., Ai G., Liu D., Zhu B. (2020). Time Series Prediction for the Epidemic Trends of COVID-19 Using the Improved LSTM Deep Learning Method: Case Studies in Russia, Peru and Iran. Chaos Solitons Fractals.

[77] Chimmula V.K.R., Zhang L. (2020). Time Series Forecasting of COVID-19 Transmission in Canada Using LSTM Networks. Chaos Solitons Fractals.

[78] Raj N., Brown J. (2021). An EEMD-BiLSTM Algorithm Integrated with Boruta Random Forest Optimiser for Significant Wave Height Forecasting along Coastal Areas of Queensland, Australia. Remote Sens..

[79] Rashed E.A., Hirata A. (2021). One-Year Lesson: Machine Learning Prediction of COVID-19 Positive Cases with Meteorological Data and Mobility Estimate in Japan. Int. J. Environ. Res. Public Health.

[80] Wang Y., Wang H., Zou D., Fu H. (2021). Ship Roll Prediction Algorithm Based on Bi-LSTM-TPA Combined Model. J. Mar. Sci. Eng..

[81] Stevenson F., Hayasi K., Bragazzi N.L., Kong J.D., Asgary A., Lieberman B., Ruan X., Mathaha T., Dahbi S.-E., Choma J. (2021). Development of an Early Alert System for an Additional Wave of COVID-19 Cases Using a Recurrent Neural Network with Long Short-Term Memory. Int. J. Environ. Res. Public Health.

[82] Satu M.S., Howlader K.C., Mahmud M., Kaiser M.S., Shariful Islam S.M., Quinn J.M.W., Alyami S.A., Moni M.A. (2021). Short-Term Prediction of COVID-19 Cases Using Machine Learning Models. Appl. Sci..

[83] Zisad S.N., Hossain M.S., Hossain M.S., Andersson K. (2021). An Integrated Neural Network and SEIR Model to Predict COVID-19. Algorithms.

[84] Awwad F.A., Mohamoud M.A., Abonazel M.R. (2021). Estimating COVID-19 cases in Makkah region of Saudi Arabia: Space-time ARIMA modeling. PLoS ONE.

[85] Tandon H., Ranjan P., Chakraborty T., Suhag V. (2020). Coronavirus (COVID-19): ARIMA based time-series analysis to forecast near future. arXiv.

